# A Review of the Science of Colorful, Plant-Based Food and Practical Strategies for “Eating the Rainbow”

**DOI:** 10.1155/2019/2125070

**Published:** 2019-06-02

**Authors:** Deanna M. Minich

**Affiliations:** ^1^University of Western States, 2900 NE 132nd Ave, Portland, OR 97230, USA; ^2^Institute for Functional Medicine, 505 S 336th St #600, Federal Way, WA 98003, USA

## Abstract

Over the past decades, thousands of published studies have amassed supporting recommendations to consume fruits and vegetables for physiological and psychological health. Newer research has emerged to suggest that these plant-based foods contain a plethora of not only vitamins and minerals, but perhaps, most importantly, phytonutrients. These phytonutrients have known pleiotropic effects on cellular structure and function, ultimately resulting in the modulation of protein kinases and subsequent epigenetic modification in a manner that leads to improved outcomes. Even though eating fruits and vegetables is a well-known feature of a healthy dietary pattern, population intakes continue to be below federal recommendations. To encourage consumers to include fruits and vegetables into their diet, an “eat by color” approach is proposed in this review. Although each individual food may have numerous effects based on its constituents, the goal of this simplified approach was to identify general patterns of benefits based on the preponderance of scientific data and known mechanisms of food-based constituents. It is suggested that such a consumer-oriented categorization of these plant-based foods may lead to greater recognition of their importance in the daily diet throughout the lifespan. Other adjunctive strategies to heighten awareness of fruits and vegetables are discussed.

## 1. Introduction

While there continues to be debate about the inclusion of meat, dairy, grains, and legumes in a healthy diet, there would seem to be little disagreement in the scientific community that eating fruits and vegetables is beneficial for one's health. Eating plant-based foods is part of many diverse dietary patterns, including the well-studied Mediterranean diet [1], vegan and vegetarian approaches, the hunter-gatherer (Paleolithic) diet [2], and even the less well-studied, ketogenic diet [3]. The quantity and quality of in vitro, animal, and clinical data over several decades suggest that intake of fruits and vegetables is associated with reducing chronic disease risk, such as cardiovascular disease, diabetes, cataracts, cancer, dementia, obesity, and others [4–7].

The search strategy for this review article was to start with a scientific literature review of the health benefits of fruits and vegetables, along with the predominant issues surrounding deficiencies in intake. Secondly, the goal was to organize the findings into a categorical system for ease of understanding and application.

### 1.1. Phytonutrient Gap

Despite the widely known health benefits of consuming fruits and vegetables, low intakes are historically consistent, with recent data from the 2015 Behavioral Risk Factor Surveillance System indicating that most adults (particularly men, young adults, and those living in poverty) consume insufficient amounts [8]. Only nine percent and twelve percent of American adults met the recommendations for vegetables and fruits, respectively [8]. Moreover, a report [9] based on food consumption data from the National Health and Nutrition Examination Survey (NHANES) conducted in 2003-2004 and 2005-2006 found that eight out of ten Americans fall short in every color of phytonutrients (referred to as a “phytonutrient gap”), especially in the color category of purple/blue foods (88% of people neglected to meet their daily serving).

Over the years, several opinion leading health organizations such as the American Institute for Cancer Research [10], the American Heart Association [11], and the USDA Food and Nutrition Service [12] have advocated eating “the rainbow” of healthy food-based colors. The 2015–2020 Dietary Guidelines [13] emphasize a healthy eating pattern across the lifespan that encourages variety and nutrient density across color categories, especially dark-green and red and orange vegetables. Dark-green vegetables are cited as good sources of vitamin K, while the red and orange vegetables are recognized for their vitamin A content. Whole fruits (fresh, canned, frozen, and dried forms) and 100% fruit juice are also included. Federal government recommendations depend on gender and age for intake but are generally 1·1/2-2 cup equivalents of fruit and 2·1/2-3 cup equivalents of vegetables daily [14]. Reduced rates of many common cancers have been associated with the equivalent of 400–600 grams daily of fruits and vegetables [7].

### 1.2. Newer Documented Benefits

It has been known for some time that the ingestion of plant foods is strongly correlated with reduction of chronic disease [5]. Newer research suggests that diets high in anti-inflammatory plant compounds such as polyphenols and other phytochemicals may help to offset pollutant toxicity [15, 16]. Thus, consuming a diet rich in plants may help buffer one's susceptibility to disease risks associated with exposure to toxic pollutants in the environment [15, 17].

Another less-recognized aspect to increased fruit and vegetable consumption is that of psychological benefit. Eating fruits and vegetables was shown to have a favorable impact on psychological well-being in 12,385 Australian adults studied longitudinally over twenty-four months using a validated questionnaire to assess overall life satisfaction [18]. Findings revealed that increasing fruit and vegetable intake (for up to eight portions daily) was positively associated with happiness, life satisfaction, and well-being, to the extent that the improvements were equal in measure to the psychological impact of transitioning from unemployment to employment. Similarly, in a large population sample of 60,404 middle- and older-aged adults [19], food intake and psychological distress assessed over almost three years of follow-up indicated that baseline fruit and vegetable intake at a certain threshold was associated with a lower prevalence of psychological distress.

In addition to reducing psychological distress and enhancing psychological well-being, curiosity, happiness, and creativity appeared to be changed in those eating fruits and vegetables. In a study of 405 young adults [20], researchers at the University of Otago in New Zealand found that those who ate more fruits and vegetables over thirteen consecutive days reported greater flourishing in daily life as assessed by higher levels of well-being, intense feelings of curiosity, and creativity, compared with young adults who ate less fruits and vegetables. These effects were not limited to the extended duration of intake. On days when young adults ate more fruits and vegetables, there was a corresponding increase in the defined markers of flourishing compared with days when they ate less fruits and vegetables. Finally, in a smartphone-based assessment logging, 1,044 completed eating episodes, and it was found that, of fourteen different main food categories, vegetable consumption contributed the largest share to eating happiness measured across eight days [21].

### 1.3. Revisiting Mechanisms

Understanding the role of complex bioactives from foods in chronic disease is difficult, considering it has been estimated that there are more than 25,000 bioactive food constituents [22]. Additionally, phytonutrients are pleiotropic and have multiple effects on cellular physiology, especially in the area of inflammation, insulin sensitization, and stress response [23, 24]. Newer research now suggests that phytonutrients play significant roles beyond their protective, antioxidant activity [25]. They have been shown to have functional and structural capacities, in addition to being cell signaling agents and messengers and modifying telomerase activity, as well as partaking in epigenetic changes through histone modification and demethylation [26, 27]. Due to their ability to address multiple mechanisms simultaneously, phytonutrients may be especially helpful in chronic diseases. For example, polyphenols have been suggested to be a potential nutraceutical intervention in type 2 diabetes [28], where there are several dysfunctional processes related to glucose and lipid metabolism that impact a number of body systems.

### 1.4. Phytochemical Index (PI)

A longstanding challenge has been quantifying the complex array of phytochemicals in the diet. While that continues to remain a research hurdle, there are now other valuable metrics that can be used. The USDA Nutrient Database [29] now includes food measurements for flavonoids, proanthocyanidins, isoflavones, and carotenoids, which cumulatively give a better estimate of a food's phytochemical content.

Phytochemical index (PI) is a relatively recent term, introduced in the cited scientific literature by McCarty in 2004 [30], and is defined as “the percent of dietary calories derived from foods rich in phytochemicals.” As outlined in the article, those calories would be from several select plant-based foods, including fruits, vegetables (excluding potatoes), fruit/vegetable juices, legumes, whole grains, nuts, seeds, soy products, wine, beer, and cider, and foods derived therefrom. Refined oils, sugars, and grains, along with potato products, hard liquors, and animal products, would be excluded from the index. While still a general estimate, the PI could be a helpful marker in epidemiological studies. It might be reasonable to envision such a marker in a smartphone application whereby a consumer could input their food intake to get a corresponding PI value. Moreover, as the PI concept evolves, it is foreseeable that it could be correlated directly to the colors of food.

Since PI was introduced in 2004, it has been used as a research measure in a variety of studies. PI has been shown to be inversely associated with body mass index, waist circumference, waist-to-hip ratio, and plasma oxidative stress [31]. A higher dietary PI was shown to have favorable effects on prevention of weight gain and reduction of body adiposity in adults [32], along with improved lipids [33, 34], and lowered risk of hypertension [35] and breast cancer [36].

As the field of personalized nutrition evolves along with a better understanding of the mechanisms of phytonutrients, there may be possibilities to do more selective phytoprofiling or targeting of conditions to certain plant-based agents for their disease-modulating effects [37].

### 1.5. Botanical Diversity and Color Density

Botanical diversity and food variety are relevant topics in the field of phytonutrients. In a recent review, Pruimboom and Muskiet [38] discussed the disparity between the plant diversity of *Homo sapiens'* diet of over 135,00 years ago at over 3000 species compared to the modern-day diet of which 400 plant species are gathered, but only more than 100 are utilized for food. Research suggests that greater variety of fruits and vegetables may have more significant impact on health markers like blood pressure, oxidative damage, and risk of falls than a less-varied diet [39–42]. In an excellent review article making a case for food diversity for the gut microbiome, Toribio-Mateas [43] recommends a “50-food challenge” chart to log intake of fresh fruits, vegetables, herbs, and spices over a 7-day period. The intended goal is to help individuals track their eating pattern for the benefit of providing a wide range of prebiotic compounds, especially polyphenols [44], from plant-based foods to feed a vast spectrum of bacteria. Indeed, even small amounts of spices have been shown to have prebiotic potential for the gut bacteria, indicating the significance of concentrated sources of phytonutrients [45].

Most plant-based foods are known to contain more than one colorful pigment, which typically corresponds to a phytonutrient or phytonutrient category, e.g., orange/beta-carotene, green/chlorophyll, and purple/flavonoids. Since a healthy eating pattern involves both a varied array (“nutrient diversity”) and dense concentration of nutrients (“nutrient density”) [46], it might be worthwhile to assess the different phytonutrient pigments contained in one food as a way of eating more “color density.” Those foods which have more than one class of phytonutrient and perhaps more corresponding colors (“greater color density”) would be those which would be most desirable for inclusion in the diet. Food listed according to their color density index is proposed in Table 1.

### 1.6. Taking a Qualitative (“Eat by Color”) Approach to Increasing Fruit and Vegetable Intake

While consuming recommended quantities of fruits and vegetables continues to be difficult for most people, it might be plausible to take a qualitative color rather than a quantitative servings approach. The concept of eating the rainbow of healthful foods would seem to be an effective strategy for assisting people in improving their diet. It can be implemented by all ages through a variety of methods. For easy reference and remembering, the importance of getting each color may be associated with some general related health benefits [7].

Preliminary research suggests that there may be relevance to the colors of fruits and vegetables and their effects in the body. For example, Mirmiran et al. [48] examined whether the colors of fruits and vegetables were associated with cardiometabolic risk factors in 1,272 adults over three years. Based on food frequency questionnaires, demographics, anthropometrics, and biochemical measures, it was found that higher intake of red/purple fruits and vegetables were related to lower weight and abdominal fat gain, and yellow, green, and white fruits and vegetables were associated with lipid parameters.

Moreover, in a Dutch prospective study over ten years, it was found that higher intakes of white fruits and vegetables were inversely associated with incident stroke. For each twenty-five gram per day increase in white fruits and vegetables (e.g., apples and pears), there was a 9% lower risk of stroke [49]. Along similar lines, the same research group found that, with each twenty-five gram per day increase in the intake of deep orange fruits and vegetables, there was an inverse association with coronary heart disease (CHD) [50]. Of these orange foods, carrots were the largest contributor (60%) with a 32% lower risk of CHD.

In this article, each of the different colors of foods will be reviewed for their health properties for specific organ systems or functions. While there is no exclusive classification of color for their physiological activities, these are general patterns based on scientific research for the ease of establishing a learning system and “art of eating” for the average consumer. More specifically, each color category, associated corresponding foods, phytonutrient content, and conferred benefit(s) were determined based on the preponderance of research publications. Thus, for the purpose of ease in categorization, this review article follows these criteria:

- *Red Foods and Inflammation*. High in antioxidants and red-food carotenoids (e.g., astaxanthin and lycopene), anti-inflammatory properties, and immune system modulation (e.g., vitamin C)
- *Orange Foods and Reproductive Health.* Abundant in carotenoids, endocrine-regulating activities, and role in fertility through support of processes such as ovulation
- *Yellow Foods and Digestion.* Rich in fibers to support a complex microbiome and assist in maintaining gastrointestinal health through gastric motility and/or digestive secretions
- *Green Foods and Cardiovascular Health*. High in a variety of nutrients for cardiovascular health, such as vitamin K, folate, magnesium, potassium, and dietary nitrates
- *Blue-Purple Foods and Cognition.* Polyphenol-rich foods to assist with learning, memory, and mood (flavonoids, procyanidins (monomeric and oligomeric form), flavonols (i.e., kaempferol, quercetin, and myricetin), phenolic acids (mainly hydroxycinnamic acids), and derivatives of stilbenes)

Summaries of the colors, nutrients, and physiological effects can be found in Table 2, while specific foods, their nutrients, and health benefits are in Table 3. Although this review uses generalized concepts about color, phytonutrients, and foods, it is important to remember that there are thousands of (phyto)nutrients present in food. The interaction between different phytochemicals that can be found inherently in the whole, plant-based food, and their interactions, is not accounted for in this overview. Certainly, there can be interactions within the food itself as well as the food with the gut microbiome; however, for the purposes of cultivating improved dietary intake of plant-based foods as the aim of this paper, these details were not addressed.

## 2. Red Foods and Inflammation

Red-colored fruits and vegetables are included in Table 2. Red-colored foods tend to be high in certain (phyto)nutrients that may confer antioxidant, anti-inflammatory, and immune-modulating activities such as ascorbic acid, lycopene, astaxanthin, fisetin, and the wider class of anthocyanins. Chronic inflammation is closely associated with a dysfunctional and dysregulated immune response, ultimately resulting in a wide variety of conditions such as cancers, neurological abnormalities, cardiovascular diseases, diabetes, obesity, pulmonary diseases, immunological diseases, and other life-threatening conditions [51].

Red-colored foods such as acerola cherry, rosehips, red bell pepper, and tomatoes also tend to be some of the highest vitamin C-containing foods [29]. Vitamin C (ascorbic acid) is well known for its effects on the immune system, and in states of increased inflammation, vitamin C levels tend to decrease in the body [52]. Several studies in cell, animals, and humans have suggested that red-colored foods and/or their isolated constituents [53] may assist with reducing systemic inflammation and bolstering immune status by reducing infections, including watermelon [54], apples [55–61], cherries [61–63], cranberries [57, 64, 65] pomegranate [66–70], and raspberries [71–73].

### 2.1. Tomatoes

Tomatoes have been widely studied in a variety of formats, from raw tomatoes to tomato juice, and even further into isolated tomato-derived phytonutrients like lycopene. They are especially known for their abundant levels of vitamin C, flavonoids (e.g., fisetin), and carotenoids (e.g., lycopene) [74]. Since they are part of the Solanaceae botanical family, there is commentary by consumer-directed websites and organizations that their alkaloid content may be inflammatory to individuals who are sensitive to those compounds.

An animal study [75] concluded that both lycopene and tomato powder supplementation given separately were equally effective in reducing inflammatory and metabolic issues that arise with a high-fat diet. Both supplemental formats helped to reduce inflammatory and lipid markers, mainly through a reduction in the phosphorylation levels of IkB and p65. A group of 106 overweight or obese female students at the Tehran University of Medical Sciences were randomly assigned either 330 ml of tomato juice or water per day for twenty days. Compared with the control group and with baseline, serum concentrations of IL-8 and TNF-*α* decreased significantly in overweight and obese female subjects [76]. Other studies using tomato juice [77] or tomato-based drinks [78] have shown beneficial effects on inflammation. In another study with tomato juice, individuals with metabolic syndrome had a significant improvement in inflammation status and endothelial dysfunction after having tomato juice four times a week over a period of two months compared with the control group [79]. Specifically, tomato products consumed with a high-fat meal were effective at attenuating postprandial lipemia-induced oxidative stress and associated inflammatory response (notably, the rise in IL-6) in healthy individuals [80].

Tomato intake in any form, whether as raw tomatoes, tomato sauce, or tomato sauce with refined olive oil, decreased plasma total cholesterol, triglycerides, and several cellular and plasma inflammatory biomarkers, and increased plasma HDL cholesterol and IL-10 concentrations [81]. However, the addition of the oil to the tomato sauce caused greater changes of plasma IL-6 and vascular cell adhesion molecule-1 (VCAM-1) and lymphocyte function-associated antigen-1 (LFA-1) from T-lymphocytes and CD36 from monocytes than after the other tomato interventions.

Overall, studies would suggest that tomato-based products, particularly when included with a meal, may offset inflammatory markers related to cardiometabolic health and oxidative stress.

### 2.2. Strawberries

Strawberries, a rich source of anti-inflammatory polyphenols such as anthocyanins, have been shown to reduce postprandial meal-induced increases in inflammation and oxidative stress in fourteen overweight health adults, particularly when the strawberry drink was consumed *before* the meal [82]. Schell et al. [83] found that obese adults with knee osteoarthritis drank a reconstituted freeze-dried strawberry beverage (50 grams daily) for twelve weeks, and it was more effective at reducing serum biomarkers of inflammation and cartilage degradation, IL-6, IL-1*β*, and matrix metalloproteinase-3 (MMP-3), compared with the control beverage.

Strawberry supplementation also significantly reduced constant, intermittent, and total pain, which led to the researchers concluding that dietary strawberries have significant analgesic and anti-inflammatory effects in obese adults with established knee osteoarthritis. Overweight adults (*n*=24) consumed a high-carbohydrate, moderate-fat meal accompanied by either a strawberry or a placebo beverage in a crossover design [84]. The strawberry beverage significantly lessened meal-evoked postprandial inflammation as measured by high-sensitivity C-reactive protein (hs-CRP) and IL-6, in addition to reducing postprandial insulin response.

Thirty-six subjects with type 2 diabetes were randomly divided into two groups [85]. The treatment group consumed two cups of freeze-dried strawberry beverage (50 g of freeze-dried strawberry is equivalent to 500 g of fresh strawberries) or macronutrient-matched placebo powder with strawberry flavor daily for six weeks in a randomized double-blind controlled trial. Freeze-dried strawberry supplementation significantly decreased CRP and malondialdehyde at six weeks compared to the baseline.

In a crossover design, fourteen women and ten men were randomized to a six-week strawberry or placebo beverage followed by a high-carbohydrate/fat meal with assessments for six hours postprandially [86]. High-carbohydrate/fat meal responses after six weeks of the strawberry beverage showed significantly attenuated inflammatory markers compared with placebo. Specifically, consumption of the strawberry beverage resulted in lower postprandial PAI-1 concentrations, especially at six hours. IL-1 *β* was also decreased in the strawberry group, and IL-6 increased significantly from baseline to six hours after the meal, following the placebo but remained relatively flat following the strawberry beverage from fasting to six hours. To summarize, strawberries, primarily as a freeze-dried beverage, appears to mitigate the inflammatory response over time and postprandially.

### 2.3. Beets

Beets provide a complex array of nutrients, but the beetroot itself is especially rich in a class of compounds known as betalains [87]. Betalains have been heralded as important considerations in chronic diseases involving inflammation, oxidative stress, and dyslipidemia [87]. A small clinical trial demonstrates the efficacy of the beet, prepared as either a juice or cooked, for reducing inflammation in hypertensive individuals. Specifically, hypertensive subjects who took either raw beet juice or cooked beet in a crossover design demonstrated that both forms of beetroot were effective in reducing systemic inflammation as assessed by intracellular adhesion molecule-1 (ICAM-1), VCAM-1, hs-CRP, IL-6, E-selectin, and TNF-*α* (*P* < 0.05) [88].

## 3. Orange Foods and Reproductive Health

Orange-colored fruits and vegetables are listed in Table 2. Orange-colored plant foods share common properties with the red-colored ones with respect to their antioxidant capacity. The primary difference is the carotenoids associated with this color class of foods, such as beta-carotene and beta-cryptoxanthin. Carotenoid compounds are fat-soluble antioxidants, stored in subcutaneous fat and in adipose tissue. While carotenoids can be ubiquitously found throughout the body due to the widespread occurrence of adipose tissue, they can be allocated to different parts of the body for particular functions [89].

There appears to be localization of specific carotenoids in certain parts of the body related to hormones and reproductive health, most likely due to their antioxidant nature [90, 91]. Oxidative stress is associated with infertility for both men and women [92]. Carotenoids may be especially important in ovaries [93]. Czeczuga-Semeniuk and Wolczynski [94] found the presence of up to fourteen different carotenoids (e.g., beta-carotene, beta-cryptoxanthin, echinenone, and hydroxyechinenone) in the ovarian tissue of 100 women operated on for ovarian tumors.

Although it has not been confirmed in humans, animal research in goats [95–98] suggests that even short-term beta-carotene can enhance or modulate ovarian function and progesterone synthesis. Furthermore, beta-carotene may have endocrine-stimulating or modulating effects as shown in prepubertal goats [99–101], given beta-carotene supplementation compared with a control group: positive changes in blood biomarkers such as total protein [99], cholesterol [99], glucose [99], insulin [100], and triiodothyronine [101] were noted. In support of the concept emerging from studies in goats that beta-carotene may influence the endocrine system, a longitudinal study [102] in 1106 men and women followed for three years showed that greater intake of dietary carotenoids, particularly those found in orange foods, such as beta‐carotene and beta‐cryptoxanthin, was associated with a reduced risk of insulin resistance.

In cattle, the highest beta-carotene levels in the plasma, corpus luteum, and follicular fluid were found during pregnancy when there is maximal luteal function, and the beta-carotene level of the corpus luteum was significantly correlated with the weight and diameter of corpus luteum [103]. While there is a paucity of human data, previous studies have indicated that women with endometriosis have lower intake of vitamin A than women without endometriosis [104]. Certain carotenoids, such as beta-carotene, are provitamin A compounds, and therefore, may be of use. Supplementation with beta-carotene and other antioxidants in women has shown to reduce time to pregnancy in couples treated for unexplained fertility [105].

Carotenoids are also important for male fertility. Sperm is susceptible to oxidative damage from the reactive oxygen species they generate, together with the fact that they have a high polyunsaturated fat content and a reduced capacity to repair DNA damage [106]. Beta-carotene was found to be associated with sperm concentration in healthy, nonsmoking men [107]. In another study [108], beta-carotene was one of the three antioxidants that significantly decreased in seminal plasma of immunoinfertile men as compared to levels in fertile men.

### 3.1. Wild Yam (*Dioscorea*)

In traditional medicine, wild yam is widely used to treat menopausal symptoms, most likely due to its phytoestrogen content and corresponding ability to stimulate ovarian estradiol synthesis [109–111]. In one study [112], twenty-four healthy postmenopausal women replaced their staple food of rice with 390 grams of yam or sweet potato (control) in two of the three meals per day for thirty days. Yam ingestion led to increases of 26% in serum concentrations of estrone, while urinary concentrations of the genotoxic estrogen, 16alpha-hydroxyestrone, decreased by 37%. Along similar lines, a variety of Chinese yam (*Dioscorea opposite* Thunb.) was shown to have estrogenic effects in vitro and in vivo [113]. Although studies are limited in food form, wild yam products are commonly used in the dietary supplement industry for enhancing progesterone levels.

### 3.2. Carrots (*Daucus carota*)

Carrots (*Daucus carota*) contain alpha- and beta-carotene [114], and extracts may have (phyto)estrogenic activity [115, 116] or be associated with estrogen metabolism [117]. While the mechanism(s) remain(s) unknown, epidemiological studies suggest that dietary carrot intake is associated with lower rates of breast [118, 119] and prostate cancer [120]. While these are preliminary findings, it would seem that, based on their carotenoid (especially beta-carotene) content, there could be an implied association with ovarian health based on the animal studies listed above, perhaps related to the concentration of carotenoids in the ovary.

### 3.3. Orange Fruits

Orange fruits include the citrus family (e.g., mandarins, oranges, and tangerines) in addition to the tropical orange fruits such as papaya, peaches, and persimmons. There is a host of nutrients to be found in the different classes, ranging from vitamin C and bioflavonoids to carotenoids such as beta-carotene and beta-cryptoxanthin.

A large study [104] of 70,835 premenopausal women as part of Nurses' Health Study II demonstrated a nonlinear inverse association between higher fruit consumption, particularly for citrus fruits, and risk of endometriosis. Women who had ≥1 servings of citrus fruits/day had a 22% lower endometriosis risk compared to those consuming <1 serving/week. Beta-cryptoxanthin, a carotenoid commonly found in orange-colored fruits, was the only nutrient examined that correlated with the lower risk of endometriosis. Furthermore, Pearce and Tremellen [121] investigated the influence of diet on the onset of natural menopause in 1146 premenopausal women followed for an average of 12.5 years. They found that the age of natural menopause is closely associated with dietary intake of beta-cryptoxanthin and fruit. It was suggested that a diet containing ∼400  mcg of *β*-cryptoxanthin per day from orange-colored fruits such as mandarins, oranges, and peaches may have the potential to delay ovarian senescence by 1.3 years. Overall, there is good emerging data to suggest that orange fruits may contain the essential carotenoids for healthy reproductive function.

## 4. Yellow Foods and Digestion

Yellow foods are found in Table 2. These foods may contain a wide array of actives that benefit the gastrointestinal tract and digestion, including bioflavonoid constituents that may modify gastric microbial activity, such as *H. pylori* and the propensity towards ulcers, or even the activity of cytochrome P450 enzymes which can ultimately modify the intestinal and/or hepatic detoxification of toxic compounds. Various soluble, insoluble, and prebiotic fibers are used to impede the release of simple carbohydrates into the bloodstream, thereby lowering glycemic index. They may also provide the raw materials required as an energy substrate to be used by the gut microbiome.

### 4.1. Ginger

Ginger is a long-recognized rhizome that contains over 400 different chemical compounds, of which gingerols and shogaols are widely discussed [122]. An extensive review of the literature suggests that ginger is helpful for a variety of gastrointestinal disorders, ranging from vomiting to dyspepsia to irritable bowel syndrome [122]. Most notably, ginger has been used traditionally for nausea [123]. A study in healthy volunteers using a standardized extract of ginger and artichoke promoted gastric emptying in healthy volunteers without adverse effects [124]. Ginger stimulated gastric emptying in healthy adults [125] and gastric emptying and antral contractions in patients with functional dyspepsia, with no impact on gastrointestinal symptoms or gut peptides [126].

### 4.2. Citrus Fruits (Lemons)

One of the distinctive features of citrus fruits is that they are acidic, mostly due to their high ascorbic acid content. This low pH may be helpful for digestive health. Several studies have reported that the glycemic response to starch-rich meals can be reduced by 20–50% with acidic drinks or foods [127]. Using an in vitro model [127], it was shown that lemon juice consumed with starch-rich foods resulted in a two-time lower breakdown of starch compared with water, suggesting a strategy to reduce the glycemic impact of high-starch meals.

Furthermore, consumption of citrus fruits has been shown to be associated with reduced risk of esophageal and gastric cancers [128, 129]. Various phytonutrients within citrus fruits such as hesperidin [130], nobiletin [131], and rutin [132] have been demonstrated to be protective against gastric ulcer, suggesting that, either alone or in combination with other agents, they could be useful therapeutics for common gastrointestinal complaints [133]. Naringenin, a flavonoid found in high concentrations in yellow citrus fruits, has been reported to have several beneficial effects, one of which involves antidiabetic activity. From a mechanistic point of view, naringenin has been shown to inhibit gluconeogenesis by upregulating AMPK [134], in addition to its influence on improving metabolic disturbances as shown in ovariectomized mice [135].

### 4.3. Pineapple

Bromelain, a proteolytic enzyme found in pineapple juice, may be helpful in metabolizing undigested food remnants in the stomach [136, 137]. In one study, drinking one liter of pineapple juice daily for three days was found to be a useful strategy for dissolving food remnants in patients undergoing endoscopic procedure for removal of intragastric balloon [138]. Similarly, adding pineapple juice to a polyethylene glycol-based solution for a colonoscopic procedure improved the quality of colon cleaning [139]. While food studies are sparse, bromelain derived from pineapple is often isolated for application in dietary supplements marketed for enzymatic activity.

### 4.4. Bananas and Plantains

Depending on their degree of ripeness, bananas contain considerable amounts of indigestible carbohydrates, which could serve as prebiotic sources for the gut microflora. In one study [140], healthy women without history of gastrointestinal disease were asked to maintain their usual dietary habits for sixty days. They were randomly assigned to consume twice a day a premeal snack, either one medium banana or one cup of banana-flavored drink or one cup of water (control group). Stool samples were collected, and gastrointestinal symptoms were also recorded. Mean bifidobacteria levels were increased only in the banana group both at thirty and sixty days of intervention, although it did not reach a statistical significance. Analysis of the gastrointestinal symptoms records revealed significantly lower bloating levels in the banana group, compared to controls, at 26–35 days (*p*=0.009) and 51–60 days (*p*=0.010).

In addition to providing a source of prebiotic fiber, both plantains and bananas (and even pineapples) were highest in the serotonin content of 80 different foods tested [141]. With many neurotransmitters being formed in the gastrointestinal tract, the implications of interaction with dietary neuroactive substances remain unknown, yet an area of research that provides promise.

## 5. Green Foods and Cardiovascular Health

Green foods are listed in Table 2. Green leafy vegetables are particularly abundant in nutrients that may be beneficial for heart health, including vitamin K (phylloquinone), magnesium, potassium, naturally occurring nitrates, and folates [142, 143]. Based on findings from a meta-analysis, Pollock [144] indicated that 15.8% of cardiovascular disease (CVD) risk could be reduced by “almost every day” consumption of green leafy vegetables, which included those in the cruciferous variety. Convincing evidence from studies exists to suggest that increasing daily consumption of vegetables and fruits can reduce risk for hypertension, coronary heart disease, and stroke [145]. Green leafy diets can be high in polyphenol concentration and provide a variety in polyphenol subclasses, which may differentially affect cardiometabolic risk factors [146, 147]. Flavonoid antioxidants such as vitexin and others have cardioprotective effects and can be found in green leafy vegetables like Swiss chard [148–150].

### 5.1. Leafy Greens (Spinach)

Leafy greens provide copious nutrients for cardiovascular health, most notably, dietary nitrates.

Short-term and even long-term (14 year) trials indicate inorganic nitrate and nitrate-rich vegetables may have vascular health benefits and lead to lowered CVD mortality [151–153]. The consumption of nitrate-rich vegetables, such as several of the leafy greens like spinach, has been shown to promote nitric oxide bioavailability, reduce systemic blood pressure, enhance tissue blood flow, modulate muscle oxygen utilization, and improve exercise tolerance, thereby potentially attenuating complications associated with limited oxygen availability or transport, hypertension, and the metabolic syndrome [154, 155]. Some of the highest nitrate containing green foods include celery, cress, chervil, lettuce, spinach, and rocket [156].

Spinach contains phytochemicals that may help with its cardiovascular benefits [157], especially nitrates. In one study [158], twenty-seven healthy participants were randomly assigned to receive either a high-nitrate (spinach; 845 mg nitrate/day) or low-nitrate soup (asparagus; 0.6 mg nitrate/day) for seven days with a one-week washout period. High- vs. low-nitrate intervention reduced central systolic and diastolic blood pressure and brachial systolic blood pressure.

### 5.2. Cruciferous Vegetables

While vegetables and fruits have consistently shown benefit for reducing CVD risk, cruciferous vegetables specifically have been shown to be associated with cardiovascular health [159]. Cruciferous vegetable intake was inversely associated with reduced cardiovascular mortality [159] and subclinical atherosclerosis [160] and 15-year atherosclerotic vascular disease deaths [161] in older adult women. Cruciferous vegetables are identified by their high concentration of organosulfur compounds such as isothiocyanates and glucosinolates [160]. Sulforaphane is an isothiocyanate with recent data indicating that its favorable effects in CVD are due to its antioxidant and anti-inflammatory properties [162, 163] as well as its ability to prevent platelet aggregation and reduce thrombus formation in flow conditions [164].

## 6. Blue-Purple Foods and Cognition

Blue-purple foods are listed in Table 2. Studies indicate that blue-purple foods are helpful for cognition and mood. Favorable studies for blue-purple foods have been documented for blueberries and grapes, both of which contain health-related phytonutrients, mainly polyphenols [165–167]. In blueberries, polyphenols include flavonoids, procyanidins (monomeric and oligomeric form), flavonols (i.e., kaempferol, quercetin, and myricetin), phenolic acids (mainly hydroxycinnamic acids), and derivatives of stilbenes [165]. Grapes possess strong antioxidant activity due to the variety of phytochemicals they contain, such as phenolic acids, stilbenes, anthocyanins, and proanthocyanins (amounts vary based on the variety). They have been shown in vitro and in vivo to inhibit cancer cell proliferation, reduce platelet aggregation, and lower cholesterol [167].

### 6.1. Concord Grape Juice

Daily intake of Concord grape juice over three to four months has been shown to improve memory function in adults with mild cognitive impairment [168]. Healthy mothers who consumed twelve ounces of either Concord grape juice or an energy-matched placebo daily for twelve weeks showed significant improvements in immediate spatial memory and driving performance with the grape juice compared with placebo [168]. These effects are not limited to those who are older in age or after chronic consumption. Haskell-Ramsay et al. [169] found that 230 mL purple grape juice improved reaction time on an attention measure and increased calm ratings in twenty healthy young adults compared with a sugar-matched control. While there are many mechanisms postulated for why grape juice may be helpful for the brain, one interesting mechanism is that it seems to modulate brain-derived neurotrophic factor (BDNF) as shown in an animal study [170].

### 6.2. Blueberries

Studies with animals suggest that blueberry supplementation of the diet may help with cognitive tasks. Willis et al. [171] found that supplementing aged animals at 2% of the diet (equivalent to ½ cup per day for humans) improved performance in the radial arm water maze, the Morris water maze, a step-down inhibitory avoidance task, and a footshock-motivated 14-unit T-maze, along with reversing cognitive decline in an object recognition test.

In addition to animal studies, there are several clinical trials demonstrating that blueberry may help cognition in older adults [172]. In a study with thirteen men and twenty-four women between the ages of 60 and 75 years, freeze-dried blueberry (24 g/day, equivalent to one cup of fresh blueberries) led to significantly fewer repetition errors in the California Verbal Learning Test and reduced switch cost on a task-switching test across study visits, relative to controls. A single dose of a flavonoid-rich blueberry drink produced significant improvements in the delayed recall of a previously learned list of words just two hours after consumption in children aged eight to ten years compared with a matched control [173]. A similar study [174] in seven- to ten-year-old children found significant wild blueberry-related improvements in cognition, such as final immediate recall at 1.15 hours, delayed word recognition sustained over each period, and accuracy on high-demand cognitive trials with increased interference at three hours. In a larger clinical trial with 16,010 participants ≥70 years [175], greater intakes of blueberries and strawberries were associated with slower rates of cognitive decline, specifically equivalent to delay cognitive aging by up to 2.5 years.

Aside from cognitive measures, inclusion of blueberry in the diet may help with mood, as has been shown in children and young adults [176]. Similar to the effects found for grape juice, blueberry supplementation seems to have an effect on cognition most likely through its effects on hippocampal BDNF mRNA expression as shown in young rats [177]. In addition, berries may help to reduce inflammation and improve cell survival and neuroplasticity [178].

## 7. Practical Ways to Get More Colorful Fruits and Vegetables

The concept proposed in this paper of “eating by color” could be tracked through a questionnaire that allows an individual to check boxes each time they have fulfilled their daily requirement of a color corresponding to an acceptable plant-based food item. This type of tracking method has been used by the author with a high degree of receptivity by individuals attempting to eat a healthier diet. Counting colors rather than calories may be a more effective way to engage long-term lifestyle change, although that concept has yet to be tested. Using this simplified approach to food may also help the category of restrained eaters, who tend to have low self-esteem [179], higher stress [180], and disordered eating patterns compared with nonrestrained eaters.

There have been several methods to help individuals increase their fruit and vegetable intake (Table 4). One of them is to encourage greater variety. Ahern et al. [181] found that vegetable consumption was promoted when variety and frequency of vegetables were increased for children between six and twelve months. Furthermore, a lunch study [182] with students showed that having vegetable options leads to increased propensity to choose vegetables and results in a more balanced meal. Other ideas for greater variety could involve nutrition education in school and implementation of concepts through school gardens [183, 184].

Variety could also include different formats of foods. The variations on raw, steamed, or boiled fruits and vegetables are vast and encompass the use of spices, seasonings, and herbs, blended fruit and vegetable drinks, herbal teas, fruit-and-vegetable-infused waters, juices, and whole-food powders. In one study [185], people who did not often consume a vegetable with lunch while dining out were 1.59 times more likely to select the seasoned vegetables over steamed vegetables. Therefore, given a choice, consumers may opt for a seasoned vegetable. Furthermore, incorporating vegetable juices and powders may also be helpful for those having difficulty in making time to prepare vegetables. Drinking vegetable juices or adding in juice powders based on tomato, carrot, or spinach was shown to reduce DNA damage and strand breaks in lymphocyte DNA in healthy individuals after several weeks' intake. The carrot juice intervention led to significantly reduced oxidative base damage [186].

Another way to emphasize fruit and vegetable intake is to eat more meals at home versus in a restaurant. Eating home-cooked meals was associated with increased consumption of fruits and vegetables and greater adherence to plant-based diets such as the Mediterranean diet. Those eating from home more than five times compared with less than three times weekly consumed 62.3 grams more fruit and 97.8 grams more vegetables daily [187].

Establishing a supportive community may also be pivotal to behavior change related to fruit and vegetable contact. Role modeling by parents correlates with fruit and vegetable intake by children [188]. Social relationships have been associated with dietary behavior [189]. Conklin et al. [189] found for both men and women that less contact with friends was correlated with less variety of fruit and vegetable intake, although the trend was more significant for men.

Finally, for the younger generation, it has been suggested that bringing in elements of fruits and vegetables into video game development could be one possible strategy to increase receptivity to healthy eating [190].

## 8. Summary

In conclusion, there are numerous benefits to eating plant-based foods, especially fruits and vegetables. Since the dietary intake continues to be less than what is recommended, it is important to develop clinical strategies to consume a greater quantity of these foods. Associating each of the colors with a health benefit for ease of remembering to eat a variety of colorful foods in one such approach may help people to relate to the health properties of fruits and vegetables. Ensuring the consumption of a variety of foods will enable the individual to sample from thousands of phytochemicals that may help to offset an increased risk of chronic disease.

## Figures and Tables

**Table 1:** Color density index (CDI) chart.

#	Plant food	Red (lycopene)	Orange (beta-carotene)	Yellow (lutein/zeaxanthin)	Green (folates)	Purple (flavonoids)
1	Acorn squash		X	X	X	N/A
2	Almonds		X	X	X	X
3	Amaranth		X	X	X	N/A
4	Apricots		X	X	X	X
5	Artichokes		X	X	X	X
6	Arugula		X	X	X	X
7	Asparagus		X	X	X	X
8	Avocado (all commercial varieties)		X	X	X	X
9	Bananas		X	X	X	X
10	Basil (dried)	X	X	X	X	
11	Beets		X		X	X
12	Black beans				X	X
13	Blackberries		X	X	X	X
14	Blueberries		X	X	X	X
15	Broccoli		X	X	X	X
16	Brussels sprouts		X	X	X	X
17	Butternut squash		X		X	N/A
18	Cabbage		X	X	X	X
19	Cantaloupe		X	X	X	X
20	Carrots	X	X	X	X	X
21	Casaba melon			X	X	N/A
22	Cashews			X	X	X
23	Cauliflower			X	X	X
24	Celery		X	X	X	X
25	Chinese cabbage (pak choi)		X	X	X	X
26	Chinese cabbage (pe-tsai)		X	X	X	X
27	Chives		X	X	X	X
28	Cilantro (coriander)		X	X	X	X
29	Cranberries		X	X	X	X
30	Cucumbers		X	X	X	X
31	Eggplant		X	X	X	X
32	Endive		X		X	X
33	Feijoa	X	X	X	X	N/A
34	Fennel		X	X	X	X
35	Figs		X	X	X	X
36	Flaxseed			X	X	N/A
37	Fuji apples		X	X	X	X
38	Gala apples		X	X	X	X
39	Garlic		X	X	X	X
40	Golden delicious apples		X	X	X	X
41	Granny smith apples		X	X	X	X
42	Grapefruit	X	X	X	X	X
43	Green and red grapes		X	X	X (especially green)	X
44	Green hot chili peppers		X	X	X	X
45	Green peas		X	X	X	X
46	Green peppers		X	X	X	X
47	Green snap beans		X	X	X	X
48	Guava	X	X		X	X
49	Hazelnuts		X	X	X	X
50	Honeydew melon		X	X	X	X
51	Iceberg lettuce		X	X	X	X
52	Jalapeño peppers		X	X	X	X
53	Jicama		X		X	N/A
54	Kale		X	X	X	X
55	Kiwi		X	X	X	X
56	Kohlrabi		X		X	X
57	Leeks		X	X	X	X
58	Lentils		X		X	X
59	Mango	X	X	X	X	X
60	Medjool dates		X	X	X	X (deglet noor)
61	Millet (cooked)		X	X	X	N/A
62	Nectarines		X	X	X	X
63	Okra		X	X	X	X
64	Onion		X	X	X	X
65	Oranges (all commercial varieties)		X	X	X	X
66	Oregano (dried)		X	X	X	X
67	Papayas	X	X	X	X	X
68	Parsley		X	X	X	X
69	Pear		X	X	X	X
70	Pecans		X	X	X	X
71	Pine nuts (dried)		X	X	X	X
72	Pineapple		X		X	X
73	Pistachios		X	X	X	X
74	Plums		X	X	X	X
75	Pumpkin		X	X	X	X
76	Pumpkin seeds (dried)		X	X	X	N/A
77	Quinoa		X	X	X	N/A
78	Radishes		X	X	X	X
79	Raspberries		X	X	X	X
80	Red cabbage	X	X	X	X	X
81	Red delicious apples		X	X	X	X
82	Red hot chili peppers		X	X	X	N/A
83	Red lentils	N/A	X	N/A	X	N/A
84	Red peppers		X	X	X	X
85	Red potatoes		X	X	X	X
86	Romaine lettuce		X	X	X	X
87	Russet (white) potatoes			X	X	X
88	Rutabagas	X	X	X	X	X
89	Savoy cabbage		X	X	X	X
90	Scallions		X	X	X	X
91	Sea vegetables (kelp)		X		X	N/A
92	Serrano peppers		X	X	X	X
93	Sesame seeds (dried)		X		X	N/A
94	Shallots		X	X	X	N/A
95	Snap peas		X	X	X	N/A
96	Sour red cherries		X	X	X	X
97	Soybeans, mature seeds		X		X	X
98	Spaghetti squash		X		X	N/A
99	Spinach		X	X	X	X
100	Spirulina		X		X	N/A
101	Strawberries		X	X	X	X
102	Summer squash		X	X	X	X
103	Sunflower seeds (dried)		X		X	N/A
104	Sweet cherries		X	X	X	X
105	Sweet potato, raw		X		X	X
106	Swiss chard		X	X	X	X
107	Tangerines		X	X	X	X
108	Tomatoes	X	X	X	X	X
109	Walnuts (English)		X	X	X	X
110	Watercress		X	X	X	X
111	Watermelon	X	X	X	X	X
112	Yellow peaches		X	X	X	X
113	Yellow peppers	N/A	X	N/A	X	X
114	Yellow plantain		X	X	X	N/A
115	Zucchini		X	X	X	X

The table is in the alphabetical order and contains many of the commonly consumed vegetables, fruits, grains, legumes, and spices. One or two main phytonutrients were used to represent the colors: lycopene for red, beta-carotene for orange, lutein and zeaxanthin for yellow, folate for green, and flavonoids for purple. The majority of information comes from the USDA National Nutrient Database [29], with some of the flavonoid information derived from the USDA Database for Flavonoid Content of Selected Foods 3.1 [47]. The latter database is not as extensive as the former, so some of the foods on this table were not included in that database. These foods have an “N/A” listed under the “purple” column. There were also a few foods for which the USDA National Nutritional Database did not list the lycopene, beta-carotene, lutein, and/or zeaxanthin content, which corresponds to an N/A listed for those foods. Finally, the table only designates that there is some quantity of these phytonutrients but does not designate which are highest in the color nor which of the colors is more dominant for the particular color. Unless designated, the results are for the raw version of the foods.

**Table 2:** Color of fruits and vegetables, select phytochemicals, and physiological effects.

Color	Fruits	Vegetables	Select phytochemicals	Physiological effects
*Red*	Apples Blood oranges Cherries Cranberries Lingonberries Nectarines Pink grapefruit Pomegranate Raspberries Red currants Red pears Red plums Strawberries Watermelon	Radicchio Radishes Red beets Red bell peppers Red cabbage Red chard Red jalapeño pepper Red onion Red potatoes Tomatoes	Anthocyanins Carotenoids Ellagic acid Ellagitannins Fisetin Flavones Lycopene Phloretin Quercetin	(i) Anti-inflammatory (ii) General antioxidant activity (iii) Immune modulation

*Orange*	Apricots Blood oranges Cantaloupe Kumquat Mandarins Mangoes Nectarines Oranges Papaya Passion fruit Peaches Persimmons Tangerines	Carrots Orange bell peppers Pumpkin Sweet potatoes Turmeric Yams	Alpha-carotene Beta-carotene Beta-cryptoxanthin Bioflavonoids Carotenoids Curcuminoids	(i) Antioxidant for fat-soluble tissues (ii) Endocrine modulation (iii) Role in ovulation and fertility processes

*Yellow*	Apples (golden delicious) Asian pears Bananas Lemons Pineapple Star fruit	Corn Ginger Potatoes (Yukon) Squash (acorn, buttercup, butternut, summer, winter) Yellow bell peppers Yellow onions	Bioflavonoids Bromelain Gingerol Lutein Nobiletin Prebiotic fibers Rutin Zeaxanthin	(i) Antioxidant (ii) Enzymatic activity (iii) Gastric motility and regulation (iv) Reduce glycemic impact (v) Role in fostering a healthy gut microbiome

*Green*	Avocado Brussels sprouts Green tea Green apples Limes Olives Pears	Artichokes Asparagus Bamboo sprouts Bean sprouts Bell peppers Bitter melon Bok choy Broccoli Broccolini Cabbage Celery Cucumbers Edamame Green beans Green peas Greens (beet, chard, collards, dandelion, kale, lettuce, mustard, spinach, turnip) Okra Rosemary and other herbs Snow peas Watercress	Catechins Chlorogenic acid Chlorophyll Epigallocatechin gallate Flavonoids Folates Glucosinolates Isoflavones Isothiocyanates L-theanine Nitrates Oleocanthal Oleuropein Phytosterols Silymarin Sulforaphane Tannins Theaflavins Tyrosol Vitexin	(i) Antioxidant (ii) Blood vessel support (iii) Role in healthy circulation and methylation

*Blue-purple*	Blackberries Blueberries Boysenberries Figs Huckleberries Prunes Purple grapes Raisins	Eggplant Plums Purple bell peppers Purple cabbage Purple carrots Purple cauliflower Purple kale Purple potatoes	Anthocyanidins Flavonoids Phenolic acids Proanthocyanidins Pterostilbene Resveratrol Stilbenes	(i) Antioxidant (ii) Cognitive support (iii) Healthy mood balance (iv) Role in neuronal health

**Table 3:** Select foods, their (phyto)nutrient profile, and health benefits.

Color	Food	Some food formats researched	Basic (phyto)nutrient profile	Researched health benefits
*Red*	Tomatoes	Juice, powder, raw, sauce (prepared with and without oil)	Carotenoids (e.g., lycopene), flavonoids, vitamin C	(i) Reduction in inflammatory markers (ii) Reduction in postprandial inflammation (iii) Improvement in lipid markers

*Red*	Strawberries	Freeze-dried as beverage	Polyphenols (flavonoids, phenolic acids, tannins), vitamin C	(i) Reduction in postprandial inflammation (ii) Reduction in pain due to osteoarthritis

*Red*	Beets	Cooked, raw juice	Betalains	(i) Reduction in inflammatory markers

*Orange*	Wild yam	Cooked	Phytoestrogens	(i) Increase in estrogen and estrogen metabolites (ii) Phytoestrogenic activity

*Orange*	Carrots	Not specified	Alpha- and beta-carotene	(i) Decrease in rate of breast and prostate cancer (ii) Phytoestrogenic activity (iii) Association with estrogen metabolism

*Orange*	Orange fruits	Not specified	Bioflavonoids, beta-carotene, beta-cryptoxanthin	(i) Delay in ovarian senescence (ii) Lower risk for endometriosis

*Yellow*	Ginger	Standardized extract	Gingerols, shogaols	(i) Decrease in nausea (ii) Increase in gastric emptying

*Yellow*	Citrus (lemons)	Juice, raw	Hesperidin, nobiletin, rutin, vitamin C	(i) Protective against gastric ulcer (ii) Antidiabetic (iii) Reduction in glycemic impact

*Yellow*	Pineapple	Juice	Bromelain, serotonin	(i) Enzymatic activity

*Yellow*	Bananas	Raw	Prebiotic fiber, serotonin	(i) Increase in bifidobacteria (ii) Reduction in bloating

*Green*	Leafy greens	Raw, spinach	Chlorophyll, folate, nitrates, phylloquinone	(i) Reduction in blood pressure (ii) Increase in nitric oxide (iii) Increase in blood flow

*Green*	Cruciferous vegetables	Not specified	Glucosinolates, isothiocyanates, sulforaphane	(i) Antioxidant action (ii) Reduction in platelet aggregation (iii) Reduction in thrombus formation

*Blue-purple*	Concord grape juice	Juice	Phenolic acids, stilbenes, anthocyanins, proanthocyanins	(i) Improvement in spatial memory and performance (ii) Improvement in reaction time on attention (iii) Increase in calm ratings

*Blue-purple*	Blueberries	Beverage, freeze-dried, raw	Flavonoids, procyanidins (monomeric and oligomeric form), flavonols (i.e., kaempferol, quercetin, myricetin), phenolic acids (mainly hydroxycinnamic acids), derivatives of stilbenes	(i) Improvement in measures of cognition (ii) Benefit to mood (iii) Improvement in neuroplasticity

This table provides a summary of certain foods and accompanying animal and/or clinical research studies as discussed in this review article. Details on the studies can be found in the respective color section in the text.

**Table 4:** Practical strategies to increase daily intake of fruits and vegetables.

(i) Increase variety by having more choices available
(ii) Cook at home rather than eat out
(iii) Choose from multiple formats, such as blended fruit and vegetable drinks (“smoothie”), juices, whole-food powders, especially with a meal
(iv) Use seasoning on vegetables
(v) Select one's community and social network carefully
(vi) Employ technology (e.g., photos, apps, games)
