# Corrigendum to “A Review of the Science of Colorful, Plant-Based Food and Practical Strategies for “Eating the Rainbow””

**DOI:** 10.1155/2020/5631762

**Published:** 2020-11-28

**Authors:** Deanna M. Minich

**Affiliations:** ^1^University of Western States, 2900 NE 132nd Ave, Portland, OR 97230, USA; ^2^Institute for Functional Medicine, 505 S 336th St #600, Federal Way, WA 98003, USA

In the article titled “A Review of the Science of Colorful, Plant-Based Food and Practical Strategies for “Eating the Rainbow”” [1], conflicts of interest should have been declared because the review is directly linked to principles covered by the author's courses and books operated via Food & Spirit, LLC.
